# Supplementary figures and images for: Characterization of Probiotic Properties and Whole-Genome Analysis of Lactobacillus johnsonii N5 and N7 Isolated from Swine
Source: Microorganisms. 2024 Mar 28;12(4):672. doi: 10.3390/microorganisms12040672 (PMC11052194; doi:10.3390/microorganisms12040672)

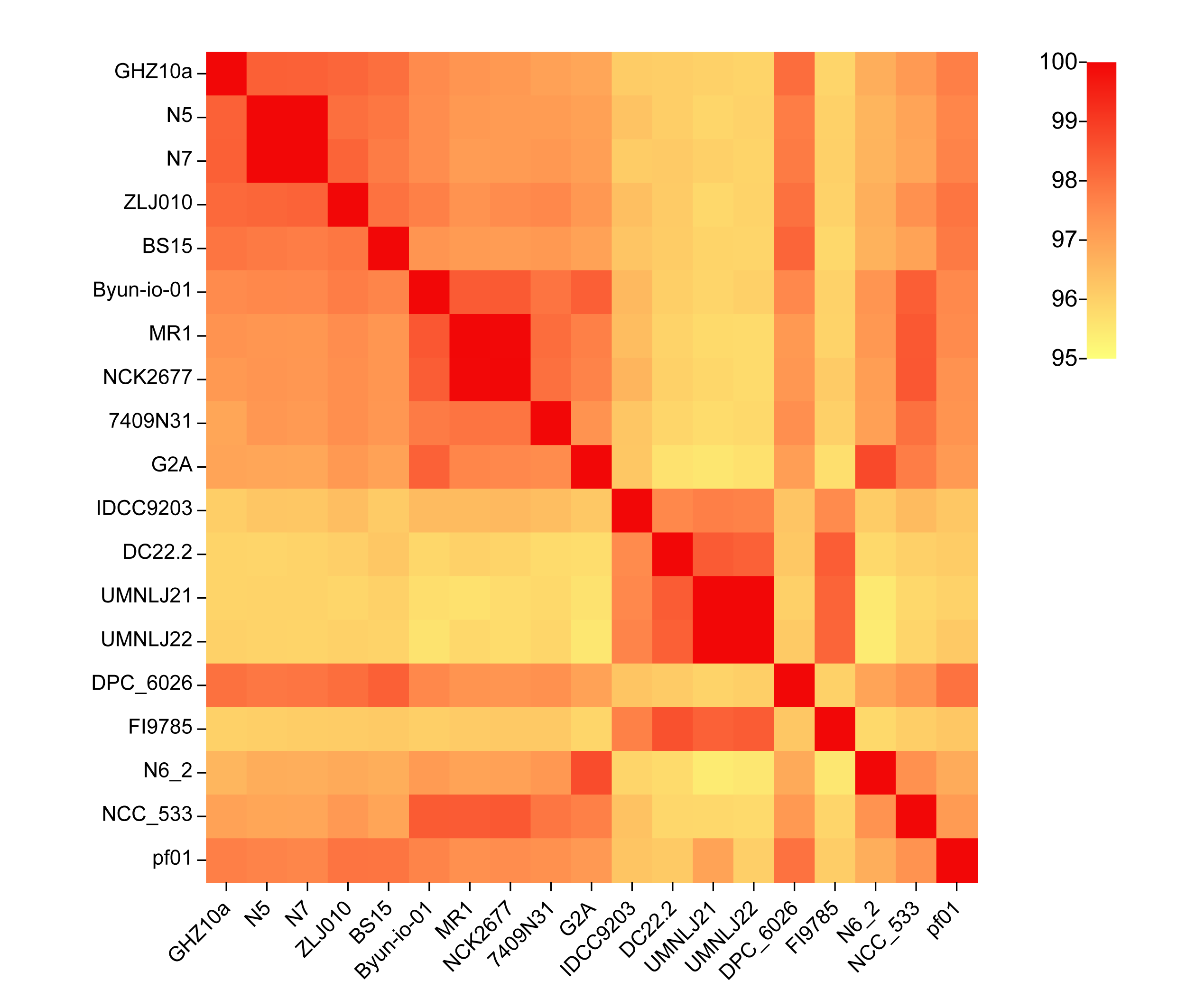

Supplement: Supplementary file 1 [file microorganisms-12-00672-s001.zip › supplementary_revise/Figure S1.tif]
